# The Network of Interactions between the Porcine Epidemic Diarrhea Virus Nucleocapsid and Host Cellular Proteins

**DOI:** 10.3390/v14102269

**Published:** 2022-10-16

**Authors:** Jianwei Zhou, Yonghui Qiu, Jie Zhao, Yongxia Wang, Ning Zhu, Dedong Wang, Yongqiu Cui, Jinshuo Guo, Tong Sun, Ying Ji, Zhi Wu, Penghui Zeng, Jingyi Li, Xufei Feng, Lei Hou, Jue Liu

**Affiliations:** 1 College of Veterinary Medicine, Yangzhou University, Yangzhou 225009, China; 2 Jiangsu Co-Innovation Center for Prevention and Control of Important Animal Infectious Diseases and Zoonoses, Yangzhou University, Yangzhou 225009, China; 3 College of Animal Science and Technology, Anhui Agricultural University, Hefei 230036, China

**Keywords:** porcine epidemic diarrhea virus, nucleocapsid protein, interaction network, GO analysis, KEGG analysis

## Abstract

Host–virus protein interactions are critical for intracellular viral propagation. Understanding the interactions between cellular and viral proteins may help us develop new antiviral strategies. Porcine epidemic diarrhea virus (PEDV) is a highly contagious coronavirus that causes severe damage to the global swine industry. Here, we employed co-immunoprecipitation and liquid chromatography-mass spectrometry to characterize 426 unique PEDV nucleocapsid (N) protein-binding proteins in infected Vero cells. A protein–protein interaction network (PPI) was created, and gene ontology (GO) annotation and Kyoto Encyclopedia of Genes and Genomes (KEGG) database analyses revealed that the PEDV N-bound proteins belong to different cellular pathways, such as nucleic acid binding, ribonucleoprotein complex binding, RNA methyltransferase, and polymerase activities. Interactions of the PEDV N protein with 11 putative proteins: tripartite motif containing 21, DEAD-box RNA helicase 24, G3BP stress granule assembly factor 1, heat shock protein family A member 8, heat shock protein 90 alpha family class B member 1, YTH domain containing 1, nucleolin, Y-box binding protein 1, vimentin, heterogeneous nuclear ribonucleoprotein A2/B1, and karyopherin subunit alpha 1, were further confirmed by in vitro co-immunoprecipitation assay. In summary, studying an interaction network can facilitate the identification of antiviral therapeutic strategies and novel targets for PEDV infection.

## 1. Introduction

Porcine epidemic diarrhea virus (PEDV) is an enveloped, single-stranded, positive-sense RNA virus belonging to the genus *Alphacoronavirus*, family *Coronaviridae*, *Nidovirales* [1]. PEDV can cause severe watery diarrhea, vomiting, and dehydration, which can result in a 100% mortality rate in suckling piglets [2]. Since 2010, PED outbreaks have been frequent on Chinese swine farms, causing enormous economic losses [3,4]. As a coronavirus, the emergence and re-emergence of PEDV is causing enormous public health problems worldwide [5,6,7].

The PEDV genome contains seven open reading frames (ORFs) encoding two polyproteins (pp1a and pp1ab), four structural proteins (spike [S], envelope [E], membrane [M], and nucleocapsid [N]), and an accessory protein (ORF3) [8,9]. Among the structural proteins, the nucleocapsid (N) plays numerous roles in virus core formation, virus assembly, virus budding, genomic RNA synthesis, chaperone activity, cell stress response to virus infection, and signal transduction [10,11]. For example, the PEDV N protein induces endoplasmic reticulum stress and activates the nuclear factor-κB signaling pathway by upregulating interleukin-8 expression and promoting viral replication [12]. In addition, PEDV N can inhibit type I interferon production mediated by IFN-I regulatory factor 3 by binding to TANK-binding kinase and by blocking the phosphorylation and nuclear translocation of IRF3 [13]. Moreover, PEDV N facilitates cell survival by binding to nucleophosmin-1 during infection [14]. This means that the PEDV nucleocapsid plays a vital role in coronavirus–host cellular interactions and can be used as a potential drug target to control PEDV infection.

Comparative research on multiple coronavirus nucleocapsid proteins can foster the development of new antiviral therapies that target the interactions between host cellular proteins and N proteins [15]. The utilization of viral proteins by various host cellular factors is important for viral replication. The PEDV genome encoding viral proteins should be multifunctional to enable it to combat host antiviral defenses, considering its limited encoding capacity. As obligate parasites, viruses depend on host–pathogen protein–protein interactions to regulate cellular biological processes for viral propagation [16]. Due to the lack of autonomous DNA polymerases, the virus relies on cellular replication machinery for its proliferation [17]. Despite initially serving as a purely structural protein, the nucleocapsid protein of coronavirus is becoming a key regulator of the virus–host cellular interface. To date, there have been several reports on the interaction between cellular proteins and the PEDV N protein, as shown by co-immunoprecipitation assays [13,14,18,19,20,21,22,23,24,25,26]. However, the broad and accurate interaction profile between PEDV N and cellular proteins still remains unknown. In addition, advanced high-throughput proteomic methods have not been used to characterize the numerous PEDV N-binding host proteins.

In this study, co-immunoprecipitation (Co-IP) combined with liquid chromatography-mass spectrometry (LC-MS) was used to map the interactome of the PEDV N protein. This process characterized 426 putative host proteins that can bind to the PEDV N in infected Vero cells. This information was used to plot a protein-protein interaction (PPI) network. Gene ontology annotation and pathway enrichment analyses revealed that PEDV N-bound host proteins are involved in many cellular pathways, such as nucleic acid binding, methylation-dependent protein binding, and RNA methyltransferase and polymerase activity. A total of 11 putative interacting proteins were selected for verification and it was confirmed that PEDV N can interact with all 11 proteins in vitro, TRIM21, DDX24, G3BP1, HSPA8, HSP90AB1, YTHDC1, NCL, YBX1, vimentin, hnRNPA2/B1, and KPNA1, although they showed different binding abilities. Therefore, the results of this study will help to identify new antiviral therapeutic targets against PEDV infection.

## 2. Materials and Methods

### 2.1. Cells and Virus

African green monkey kidney cells (Vero cells, CCL-81; American Type Culture Collection [ATCC], Manassas, VA, USA) were maintained in Dulbecco’s modified Eagle’s medium (DMEM) (Gibco, Carlsbad, CA, USA) supplemented with 10% fetal bovine serum (FBS) (S711-001S; LONSERA, Shanghai Shuangru Biology Science & Technology Co., Ltd, Shuangru, Shanghai, China). HEK293T cells (CRL-11268; ATCC, Manassas, VA, USA) were cultured in DMEM (Gibco) supplemented with 10% fetal bovine serum (FBS) (Gibco). IPEC-DQ cells, a subclone of the IPEC-J2 porcine intestinal epithelial cell line, were a courteous gift of Dr. Dongwan Yoo, College of Veterinary Medicine, the University of Illinois at Urbana-Champaign, Urbana, Illinois, USA. This cell line was cultured in RPMI 1640 media (C11875500BT; Gibco) containing 10% FBS (Gibco). Porcine epidemic diarrhea virus (PEDV) strain LZW isolate CPGEN_20140427 (accession no. KJ777678.1) was propagated and stored in our laboratory.

### 2.2. Antibodies and Reagents

Anti-β-actin (M1210-2) mouse monoclonal antibody (mAb) and anti-Myc (R1208-1), anti-FLAG (0912-1), and anti-GFP (SR48-02) rabbit polyclonal antibodies (pAbs) were purchased from Huaan Biological Technology (Hangzhou, China). Anti-GFP (B-2, sc-9996) mouse mAb for immunoprecipitation was acquired from Santa Cruz Biotechnology (Dallas, TX, USA). Anti-FLAG (F1804), and anti-Myc (05-419) mouse mAbs used for IP were obtained from Sigma-Aldrich (St. Louis, MO, USA). Anti-FLAG affinity resin (A2220) for immunoprecipitation (IP) was purchased from Sigma-Aldrich. Rabbit mAb against YTHDC1 (ab259990) and rabbit mAb against NCL (ab129200) were purchased from Abcam (Cambridge, MA, USA). Anti-PEDV N mAb was prepared and stored in our laboratory. NP-40 cell lysis buffer (50 mM Tris [pH 7.4], 150 mM NaCl, and 1% NP-40) was obtained from Beyotime (P0013F; Shanghai, China). Horseradish peroxidase (HRP)-labeled goat anti-mouse and anti-rabbit IgG antibodies were purchased from KPL (Milford, MA, USA).

### 2.3. Plasmid Construction and Cell Transfection

A DNA fragment encoding the full-length of PEDV *N* mRNA was amplified by polymerase chain reaction (PCR) using genomic complementary DNA (cDNA) of PEDV strain LZW (accession no. KJ777678.1) and then separately subcloned into vectors pCMV-FLAG-N (Clontech, Palo Alto, CA, USA) and pCMV-Myc-N (Clontech). The full-length cDNA sequences of *TRIM21* (accession no. XM_008019947.2), *DDX24* (accession no. XM_007987681.2), *G3BP1* (accession no. XM_008015062.2), *HSPA8* (accession no. XM_008021262.2), *HSP90AB1* (accession no. XM_007972399.2), *YTHDC1* (accession no. XM_007998565.2), *NCL* (accession no. XM_038001384.1), *YBX1* (accession no. XM_007979148.2), *vimentin* (accession no. XM_008002397.2), *hnRNPA2/B1* (accession no. XM_007981833.2), and *KPNA1* (accession no. XM_007985606.2) were amplified from Vero cells using specific primers, and they were subcloned separately into vector pEGFP-C3 (Clontech). The resultant plasmids were GFP-TRIM21, GFP-DDX24, GFP-G3BP1, GFP-HSPA8, GFP-HSP90AB1, GFP-YTHDC1, GFP-NCL, GFP-YBX1, GFP-vimentin, GFP-hnRNPA2/B1, and GFP-KPNA1. The detailed procedures for plasmids construction that were carried out are described in previous research [27,28,29]. The primers adopted are summarized in Table 1. HEK293T cells were grown on plates up to 70% to 90% confluency and then were co-transfected with 4.0 μg of the respectively indicated plasmids. The ExFect transfection reagent (T101-01/02; Vazyme Biotechnology, Nanjing, China) was used for HEK293T cell transfection as described in the manufactures’ protocols. The procedures for plasmid construction and cell transfection assays were conducted as described elsewhere [30,31].

### 2.4. SDS-PAGE and Western Blotting

Cell lysate extracts prepared in lysis buffer after transfection were separated using sodium dodecyl sulfate-polyacrylamide gel electrophoresis (SDS-PAGE). The samples were then transferred to nitrocellulose membranes (GE Healthcare, Chicago, IL, USA) and blocked in phosphate-buffered saline (PBS) containing 5% skimmed milk powder and 0.05% Tween 20. Membranes were then incubated with primary antibodies overnight at 4 °C, followed by incubation with HRP-labeled secondary antibodies at room temperature for 1.0 h. The membranes were then incubated with an enhanced chemiluminescence reagent (34096; Thermo Scientific, Waltham, MA, USA), and the immunoreactive protein bands were visualized using AI800 Images (Cytiva Sweden AB, Uppsala, Sweden). SDS-PAGE and WB assays were performed as previously described [30,31].

### 2.5. Co-Immunoprecipitation (Co-IP) Assays

For the co-IP assays, HEK293T cells transfected with the indicated plasmids for 48 h were lysed in NP-40 cell lysis buffer and centrifuged at 12,000× *g* for 10 min. The supernatants were then treated with protein A/G plus agarose (sc-2002; Santa Cruz Biotechnology, CA, USA) for 1.0 h at 4 °C and immunoprecipitated using anti-FLAG beads, anti-GFP mAbs, or anti-Myc mAbs. The beads or agarose were washed with NP-40 buffer and resolved using standard SDS-PAGE. Co-IP assays were performed as described previously [30,31].

### 2.6. Liquid Chromatography Mass Spectrometry (LC-MS)

Coomassie blue stained gels from co-immunoprecipitation experiments were pooled and analyzed for protein identification by liquid chromatography mass spectrometry (LC-MS) analysis in APTBio (Shanghai, China). The resulting peptides were concentrated and desalted on an EASY column (2 cm × 100 μm 5 μm-C18; 75 μm × 100 mm 3 μm-C18; Thermo Finnigan, San Jose, CA, USA) and eluted online on an analytical RP column (0.18 cm × 150 mm BioBasic-18, Thermo Electron, Waltham, MA, USA). A 60 min gradient from 4% to 50% B (solvent A: 0.1% *v*/*v* formic acid; solvent B: 0.1% *v*/*v* formic acid, 84% *v*/*v* ACN) 0 min–50 min, 50–100% B for 50 min–54 min, and 100% B for 54 min–60 min, were used. Protein searches were performed using the Mascot 2.2 software. Proteins found in the individual negative control samples were eliminated from the datasets to eliminate non-specific interactions. Proteins represented by at least two unique peptides were used for further analyses. The LC-MS procedures were conducted as described elsewhere [27].

### 2.7. Construction and Analysis of the Protein-Protein Interactions Network

All experimentally derived data sets were used to generate the PEDV N-host protein interactions network by using Cytoscape version 3.7.1. The STRING database was used to analyze interactions among the host proteins. Only interactions confirmed by direct physical binding were considered for mapping protein–protein interactions. Topological parameters and fundamental measures of the network were calculated using the network analyzer tool in Cytoscape 3.7.1. African green monkey protein–protein interaction analysis was also performed using the STRING database. In all of the networks and throughout the study, we used NCBI gene names to denote proteins to achieve a consensus in protein accession. The corresponding NCBI gene names are listed separately (Supplementary Table S2). PPIs were performed as described previously [27].

### 2.8. GO and KEGG Pathway Analyses

Gene ontology (GO) analysis was performed using Cytoscape software (version 3.7.1) with the GOclue plugin to annotate the genes in terms of cellular component (CC), biological process (BP), and molecular function (MF) based on the GO database (Supplementary Table S3). Kyoto Encyclopedia of Genes and Genomes (KEGG) enrichment analysis was performed to predict pathways based on the KEGG database (Supplementary Table S4). The GO and KEGG pathways with *p*-value < 0.05 denoted pathways with significant increases. GO and KEGG pathway analyses were completed by APTBio (Shanghai, China) and carried out as previously described [27].

### 2.9. Confocal Microscopy

IPEC-DQ cells were infected with PEDV at a MOI of 0.5 for 12 and 24 h. The cells were washed with PBS and fixed with 4% paraformaldehyde for 10 min and then permeabilized with 0.1% Triton-X 100 for 10 min at room temperature. Mouse anti-PEDN N mAb and rabbit anti-YTHDC1 and anti-NCL mAbs were used as primary antibodies, and fluorescein isothiocyanate (FITC)-conjugated goat anti-mouse IgG and Alexa Fluor 546-conjugated donkey anti-rabbit IgG were used as secondary antibodies. Cellular nuclei were stained with 10 μg/mL 4′,6′-diamidino-2-phenylindole (DAPI; 10236276001; Roche, Mannheim, Germany) to obtain images under a LSM880 laser scanning confocal microscope (Zeiss, Oberkochen, Germany).

## 3. Results

### 3.1. Identification of PEDV N Protein-Host Protein Interactions by Liquid Chromatography-Mass Spectrometry

**Figure 1:**
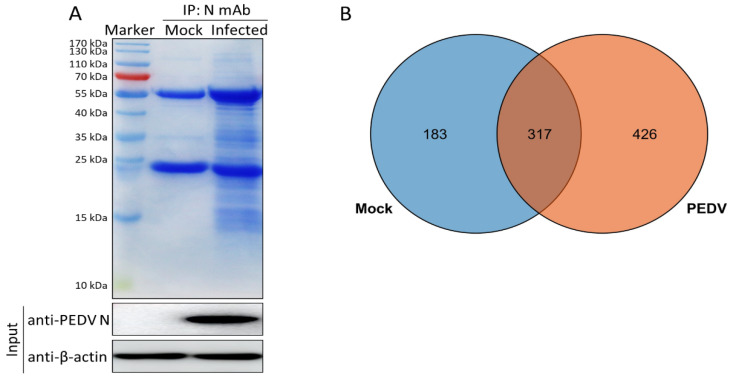
Identification of PEDV N protein-interacting cellular proteins. (**A**) Mock-infected Vero cells or cells infected with PEDV at a MOI of 0.5 were harvested at 24 hpi, and a co-immunoprecipitation assay was performed. Purified anti-N IgG. PEDV N-bound host proteins were eluted and analyzed via SDS-PAGE followed by Coomassie blue staining. Lane 1, protein molecular weight ladder; lane 2, mock-infected; lane 3, PEDV-infected. (**B**) Venn diagram of the characterized protein candidates binding to the PEDV N protein from mock-infected, and PEDV-infected cells, respectively. Blue and orange colors indicate proteins from the mock-infected, and PEDV-infected cells, respectively. Common proteins within the data sets are shown in the colored intersections. Proteins were presented as their respective NCBI gene names (Supplementary Table S1).

To identify potential host cellular protein interactions with the PEDV N protein in infected Vero cells, we performed Co-IP assays in combination with LC-MS/MS in the presence or absence of PEDV infection. Whole cell lysates were co-immunoprecipitated with purified anti-N IgG at 24 h post-infection (hpi), and Coomassie blue staining was used to visualize the host proteins bound to the PEDV N protein (Figure 1A). As a negative control, mock-infected Vero cell lysates were used to eliminate nonspecific binding. The differential bands were visualized and compared with those of the negative control. LC-MS was used to identify the cellular proteins bound to the PEDV N protein. A total of 743 host cellular candidates bound to PEDV N proteins were identified in the infected Vero cells (Figure 1B and Supplementary Table S1). Of these, 317 cellular proteins were also present in the mock cell lysates; therefore, without further interpretation, the remaining 426 specifically expressed and bound host proteins were considered to be novel cellular candidates binding to the PEDV N protein in infected Vero cells, and these proteins were subjected to further analyses (Figure 1B and Supplementary Table S2).

### 3.2. Construction of a Protein-Protein Interaction Network

**Figure 2:**
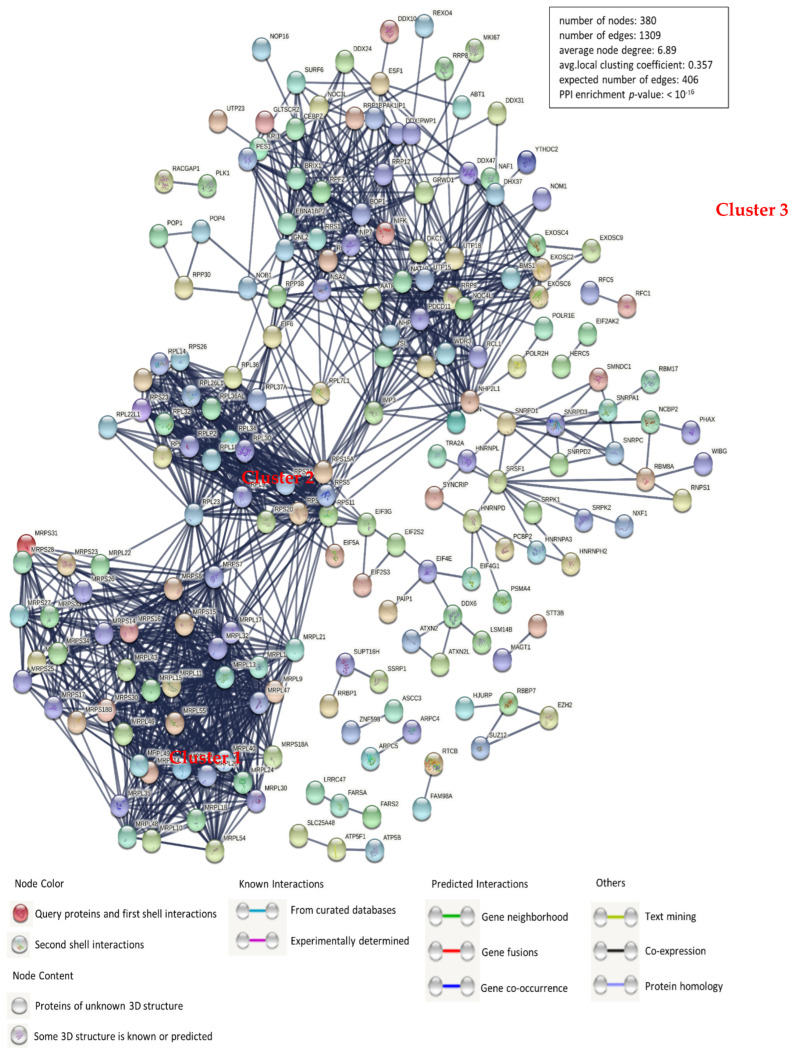
Construction and analysis of the protein–protein interaction network using the STRING database. Each edge color indicates a unique method of protein–protein interaction prediction, as indicated below the figure. A map of the interaction of PEDV N protein–interacting host proteins with the other proteins in our data was constructed and plotted using the network analyzer tool from Cytoscape software, version 3.7.1. The corresponding symbols indicating the different protein classes are shown in the figure. Proteins are represented by their respective NCBI gene names (Supplementary Table S2).

Because of the vital roles of host cellular factors, the verification of protein–protein interactions (PPIs) is a decisive aspect of molecular biology. Here, we have mapped the interaction network between PEDV N protein-bound host proteins and cellular proteins using the STRING database for network structure and function analyses (Figure 2 and Supplementary Table S2). Together with proven protein interactions, those acquired from gene fusion, co-expression, homology, and text mining were used to construct the network. The host proteins in the interaction network are predominantly divided into ribonucleoprotein complex biogenesis modulation, cellular nitrogen compound metabolism, and nucleic acid binding. There are three large and distinct clusters, cluster 1 denoting mitochondrial ribosomal proteins, cluster 2 denoting ribosomal proteins, and cluster 3 comprising those with uncharacterized concrete functions. For a constant number of nodes (380), the number of edges (1309) in the network was significantly higher than the expected number (406), indicating more interactions than expected for a group of random proteins. These results demonstrate that these proteins were divided into several roles, mainly replication and transcription.

### 3.3. Gene Ontology Annotation and Analysis

**Figure 3:**
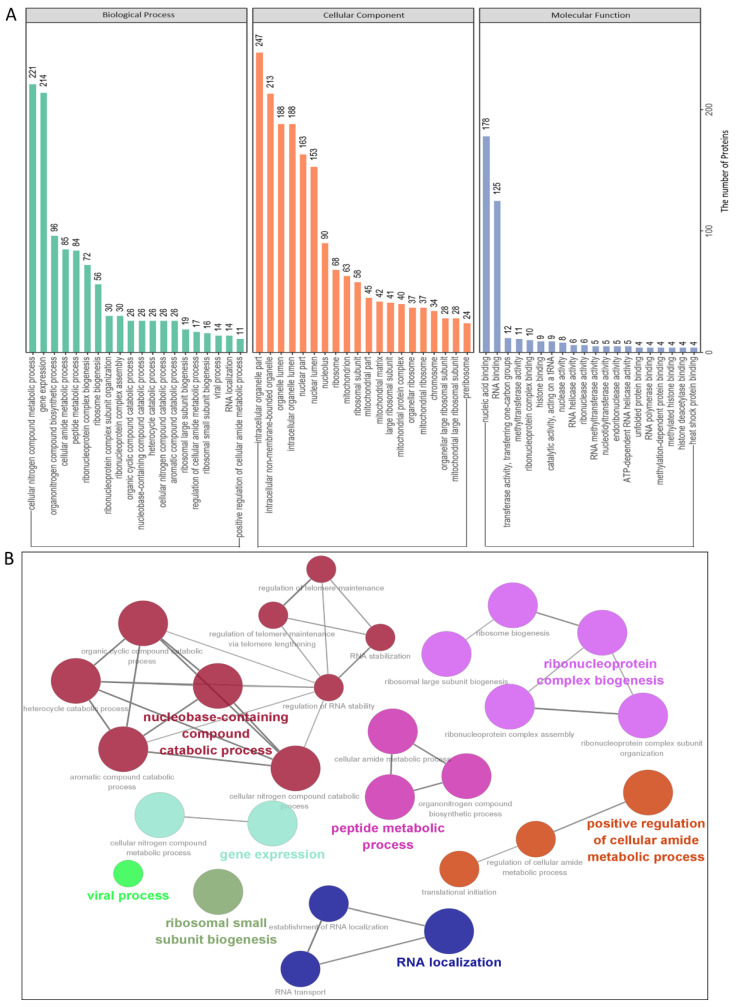
Gene Ontology analysis of the identified PEDV N protein–host interactome. (**A**,**B**) Representative over-represented GO terms of protein clusters and the GO distribution of unique proteins in the PEDV-infected cells were classified into three categories using the GOclue plugin in Cytoscape software, version 3.7.1. We have shown the significantly enriched terms based on the biological process (BP), molecular function (MF), and cellular component (CC) with *p*-values < 0.05. The Roman numerals represent the detailed GO terms, as shown in Supplementary Table S3.

To identify the cellular pathways in the PEDV N–host protein interaction network, we conducted gene ontology annotation for the proteins specifically expressed and bound to the PEDV N (Supplementary Table S2) to predict their molecular functions. GO annotation was performed for the following three categories: biological processes, molecular functions, and cellular components. Numerous biological processes, such as cellular nitrogen compound metabolism, gene expression, organonitrogen compound biosynthesis, and ribonucleoprotein complex biogenesis, were also found to be increased. In addition, nucleic acid binding, ribonucleoprotein complex binding, and RNA methyltransferase and polymerase activities were increased with regard to their activities, whereas intracellular organelles, intracellular non-membrane-bounded organelles, intracellular organelle lumen, and nuclear lumen were increased with regard to their abundance (Figure 3A,B and Supplementary Table S3). In summary, GO annotation suggested that the PEDV N protein might interact with several processes, such as cellular nitrogen compound metabolism, ribonucleoprotein complex biogenesis, gene expression, and RNA methyltransferase and polymerase activities.

### 3.4. KEGG Pathway Enrichment Analysis

**Figure 4:**
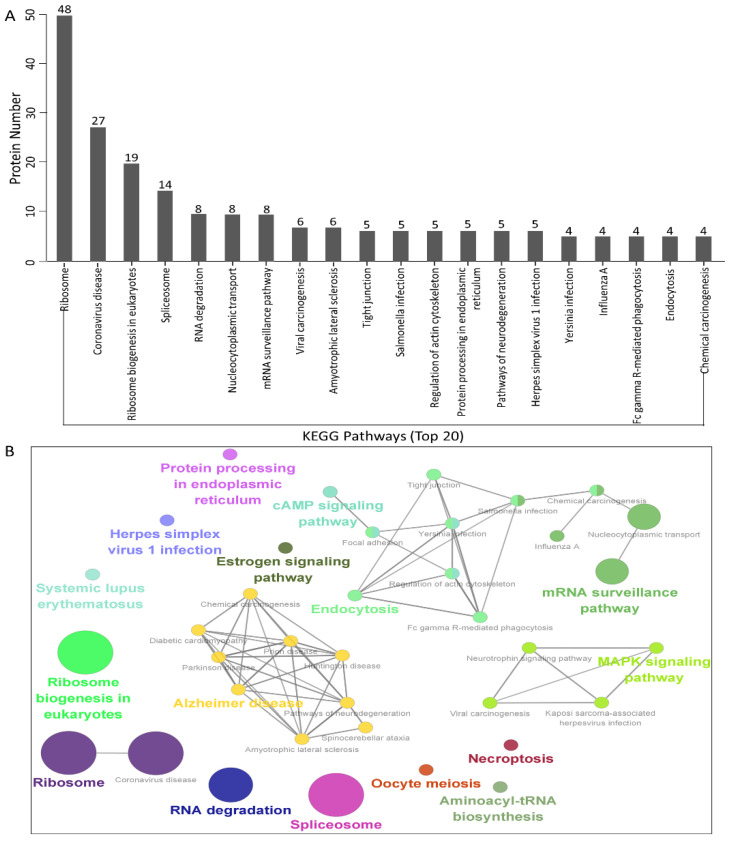
KEGG pathway enrichment analysis. (**A**,**B**) Graphs showing the enriched pathways targeted by the PEDV N protein-interacting proteins, as analyzed via KEGG functional annotation (Supplementary Table S4) using the GOclue plugin in Cytoscape software, version 3.7.1.

To establish host signal transduction pathways related to N protein-bound cellular proteins targeted by PEDV (Supplementary Table S2), we performed Kyoto Encyclopedia of Genes and Genomes (KEGG) pathway enrichment and obtained the 20 enriched pathways with the highest representation for each term. It is worth noting that most of the potential proteins were involved in the ribosomes, coronavirus disease, ribosome biogenesis in eukaryotes, spliceosome, RNA degradation, nucleocytoplasmic transport, and the mRNA surveillance pathway. KEGG enrichment analysis indicated that pathways involving ribosomes, RNA degradation, and coronavirus disease were preferentially enriched (Figure 4A,B and Supplementary Table S4). In addition, KEGG enrichment analysis suggested that these proteins may play key roles in regulating different processes, such as viral carcinogenesis and protein processing in the endoplasmic reticulum.

### 3.5. Validation of the Interactions between the Host Proteins and the PEDV Nucleocapsid Protein

**Figure 5:**
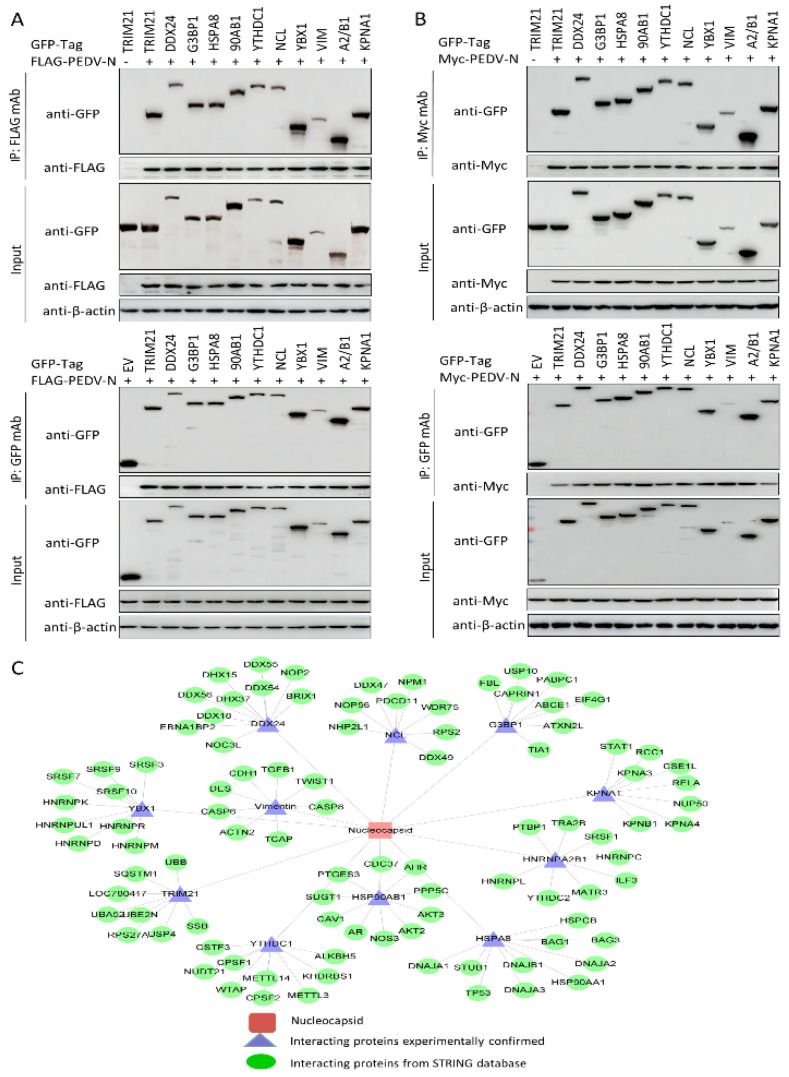
Validation of PEDV N–host protein interactions. (**A**,**B**) HEK293T cells were co-transfected with plasmids expressing either GFP-TRIM21, GFP-DDX24, GFP-G3BP1, GFP-HSPA8, GFP-HSP90AB1, GFP-YTHDC1, GFP-NCL, GFP-YBX1, GFP-vimentin, GFP-hnRNPA2/B1, or GFP-KPNA1 and plasmids expressing FLAG-PEDV-N (**A**), or Myc-PEDV-N (**B**). Among them, FLAG-PEDV-N (**A**), Myc-PEDV-N (**B**), and GFP-TRIM21 (**A**,**B**) were all co-transfected with an empty vector serving as the negative controls, while FLAG-PEDV-N (**A**), or Myc-PEDV-N (**B**) co-transfected with GFP-TRIM21 (**A**,**B**) served as positive controls. Cell lysates were immunoprecipitated with FLAG beads, or anti-GFP mAbs, or anti-Myc mAbs and then separated via SDS-PAGE. Western blotting was then performed with the corresponding primary and secondary antibodies. β-actin served as the internal loading control. (**C**) The PEDV N–host interaction network. The interaction map of PEDV N and the corresponding host proteins was constructed using Cytoscape. Proteins were classified based on their protein class. The corresponding symbols indicating different protein classes are mentioned in the Supplementary Table S5.

To further validate protein interactions using mass spectrometry, we performed in vitro Co-IP assays. Eleven host proteins from the PEDV-infected Vero cells were chosen to validate mass spectrometry data based on the proteomics parameters such as the number of peptides, the number of unique peptides, cover percent, molecular weight, pI, etc. HEK293T cells were co-transfected with FLAG-PEDV-N and either an empty vector, GFP-TRIM21, DDX24, G3BP1, HSPA8, HSP90AB1, YTHDC1, NCL, YBX1, vimentin, hnRNPA2/B1, or KPNA1 expression constructs, followed by co-immunoprecipitation using FLAG beads or anti-GFP monoclonal antibodies (mAbs). The results indicated that PEDV N protein specifically bound to TRIM21, DDX24, G3BP1, HSPA8, HSP90AB1, YTHDC1, NCL, YBX1, vimentin, hnRNPA2/B1, and KPNA1, whereas no signal was observed with the empty vector. It should be noted that the different proteins exhibited distinct binding capacities (Figure 5A). To eliminate the effect of the FLAG tag on the authenticity of the results, we performed a similar assay using Myc-PEDV-N, and the results were consistent with those of the FLAG-PEDV-N expression plasmid (Figure 5B). Thus, the results acquired from Co-IP assays verified the data from the proteomic analyses based on LC-MS. Next, we used Cytoscape to map the interaction network of the experimentally validated cellular protein-PEDV N protein interactions and the host partners of the PEDV N-binding cellular proteins *in silico* (Figure 5C and Supplementary Table S5), which may help to study the potential roles of PEDV N protein in the virus life cycle.

### 3.6. PEDV Infection Resulted in the Redistribution of YTHDC1 and NCL

**Figure 6:**
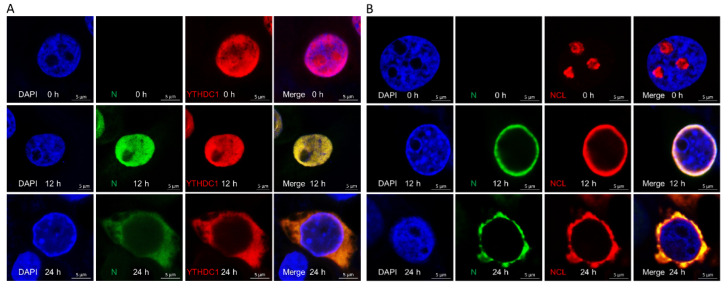
YTHDC1 and NCL relocate from the nucleus/nucleolus to the cytoplasm induced by PEDV infection. (**A**,**B**) Immunofluorescence analyses of YTHDC1 and NCL protein localization during PEDV infection. IPEC-DQ cells were infected with PEDV at a MOI of 0.5. The cells were fixed at 12 and 24 hpi and incubated with the antibodies corresponding to PEDV N, YTHDC1, and NCL followed by the FITC-conjugated goat anti-mouse IgG (green) and Alexa Fluor-546 conjugated donkey anti-rabbit IgG (red) secondary antibodies. Nuclei were stained with DAPI (blue) and then observed under a confocal microscope. Scale bar, 10 μm.

To demonstrate whether the localization of YTHDC1 and NCL proteins was altered during PEDV infection, we determined the distributions of YTHDC1 and NCL in IPEC-DQ cells by confocal microscopy. IPEC-DQ cells were infected with PEDV at a MOI of 0.5 and fixed at 12 and 24 hpi. The localization of PEDV N and YTHDC1 protein and PEDV N with NCL protein were detected by confocal microscopy, and the co-localizations of PEDV N and endogenous YTHDC1 protein and of PEDV N and NCL protein were observed in the nucleus at 12 hpi and YTHDC1 was mainly resided in the nucleus, while NCL was mainly resided in the nucleolus in mock-infected cells (Figure 6A,B). Subsequently, the subcellular localization of endogenous YTHDC1 and NCL altered at 24 hpi. Our results indicated that YTHDC1 and NCL relocated from the nucleus/nucleolus to the cytoplasm in PEDV-infected IPEC-DQ cells during the late stage. 

### 3.7. The YTHDC1 and NCL Expression Inhibited PEDV Replication

**Figure 7:**
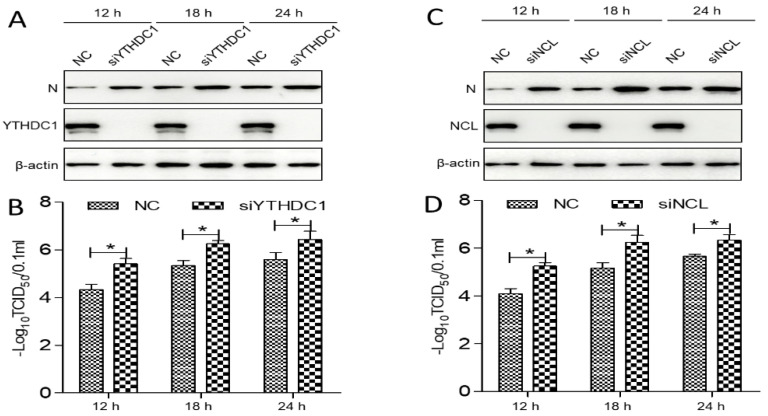
The YTHDC1 and NCL expression inhibited PEDV replication. (**A**,**C**) The *YTHDC1*-silenced *or NCL*-silenced IPEC-DQ cells were infected with PEDV at a MOI of 0.5 for 12, 18, and 24 h. The cell lysates were analyzed by immunoblotting to examine protein levels of N, YTHDC1, NCL, and β-actin. (**B**,**D**) The samples from (**A**,**C**) were used to measure PEDV replication by determining TCID_50_ values. Data are presented as means ± SD of three independent biological experiments. * *p* < 0.05.

As YTHDC1 and NCL translocated from the nucleus/nucleolus to the cytoplasm during PEDV infection, the *YTHDC1*-silenced *or NCL*-silenced IPEC-DQ cells were used to detect the level of PEDV replication. The small interfering RNAs (siRNAs) specific for *YTHDC1* (5′-GCAGGCGUGUUTTACCCUU-3′) or *NCL* (5′-GAAUUGGUGUGUCUAGGAA-3′) were designed and synthesized to knockdown YTHDC1 or NCL expression. Afterwards, these cells were inoculated with PEDV at a MOI of 0.5, and viral replication was monitored by determining virus titer and the expression level of N protein. The data showed that the expression of N was significantly elevated in *YTHDC1*-silenced or *NCL*-silenced cells compared with control cells (Figure 7A,C; *p* < 0.05). Similarly, the viral titer of PEDV also increased significantly in *YTHDC1*-silenced or *NCL*-silenced cells (Figure 7B,D; *p* < 0.05), suggesting that PEDV replication is upregulated in *YTHDC1*-silenced or *NCL*-silenced cells. Collectively, these results demonstrated that the YTHDC1 and NCL expression inhibited PEDV replication.

## 4. Discussion

Coronaviruses are widespread in humans, pigs, birds, and other mammals, causing respiratory and enteric diseases [32]. Over the past 20 years, coronaviruses have caused three major pandemics: SARS, MERS, and SARS-CoV-2. These three coronaviruses spread rapidly worldwide due to their high transmission capacity [33]. They also often have a high mortality rate. At present, the worldwide SARS-CoV-2 pandemic has aroused special interest in research related to coronaviruses in the field of virology. Emerging coronaviruses can cause serious illnesses in humans and animals; however, currently available drugs and vaccines cannot effectively control these diseases. Therefore, there is an urgent need to identify/develop effective antiviral drugs against novel viruses.

PEDV is an enveloped, single-stranded, positive-sense RNA virus within the *Coronaviridae* family. It is an emerging and re-emerging alphacoronavirus that causes acute diarrhea and vomiting and has high mortality in newborn piglets, resulting in huge economic losses for the global swine industry [1,2,3,34,35]. As obligate parasites, viruses rely completely on cellular pathways to obtain the resources needed for replication. Thus, virus–host interactions play a major role in the viral life cycle. The PEDV nucleocapsid (N) protein has multiple functions in virus core formation, virus assembly, virus budding, genomic RNA synthesis, chaperone activity, cellular stress response to viral infection, and signaling [10,11]. However, it remains unclear whether PEDV N disrupts or exploits the host machinery for viral replication. In addition, the interaction between a large number of cellular proteins and PEDV N has not been determined.

Previous studies have reported 11 of the host interacting proteins with PEDV N by other approaches [13,14,18,19,20,21,22,23,24,25,26]. Among these 11 proteins, only 3 proteins, namely TRIM21, nucleophosmin-1 (NPM1), and poly(A)-binding protein 4 (PABPC4) were confirmed in the present study. We performed the interactions with a PEDV infection (strain LZW CPGEN_20140427) at a MOI of 0.5 and harvested at 24 hpi, and these differences in PEDV strains, cell lines, and sampling times may impact the interactions. However, whether PEDV N can interact with all host proteins identified in the present study requires further validation in the future.

In this study, we characterized 426 specific host proteins that bind to the PEDV N via Co-IP and LC-MS (Figure 1). Subsequently, a PPI network was constructed (Figure 2). The gene ontology (GO) and Kyoto Encyclopedia of Genes and Genomes (KEGG) pathway analyses showed that binding host proteins are involved in a variety of biological processes, such as cellular nitrogen compound metabolism, gene expression, organonitrogen compound biosynthesis, ribonucleoprotein complex biogenesis, and RNA methyltransferase and polymerase activities (Figure 3 and Figure 4). We also verified the interactions of PEDV N with 11 potential host proteins: TRIM21, DDX24, G3BP1, HSPA8, HSP90AB1, YTHDC1, NCL, YBX1, vimentin, hnRNPA2/B1, and KPNA1 *in vitro* using Co-IP assays (Figure 5). To date, our research is the first to identify the host proteins bound to PEDV N using modern proteomic tools such as LC-MS. Bioinformatics analyses of existing datasets will help with understanding the roles of cellular proteins or PEDV N in biological pathways, viral replication, and pathogenesis.

The proteomics analysis in this study could only qualitatively identify 426 host-interacting proteins in the PEDV-infected Vero cells. In a previous paper [36], we used isobaric tags for relative and absolute quantitation (iTRAQ)-coupled LC-MS/MS to quantitatively identify the differential proteome after PCV2-classic swine fever virus coinfection and found 788 differential host proteins with fold changes in protein expression in coinfected cells. Therefore, combining both methods of target protein selection will help qualitatively and quantitatively identify the host-interacting proteins.

Several biological processes such as gene expression, biosynthesis of organonitrogen compounds, ribonucleoprotein complex biogenesis, RNA methyltransferase and polymerase activities, as well as nucleocytoplasmic transport pathway and spliceosome pathway are very important in PEDV infection processes that require special attention in future studies. Although we have characterized some of the proteins associated with these signaling pathways in this study, their precise roles remain unclear; therefore, further studies are essential. An important feature of the virus–host interaction is the manipulation of nucleocytoplasmic transport in order to create a favorable cellular environment for efficient virus replication. It is hypothesized that PEDV could hijack the nucleocytoplasmic transport pathway and cellular splicing pathway for PEDV replication. GO annotation and KEGG enrichment analyses showed that proteins associated with the spliceosome pathway were increased (Figure 3 and Figure 4). In the present study, some proteins of nucleocytoplasmic transport pathways, including KPNA1, KPNA2, KPNA4, and importin β1 (KPNB1), were found to interact with PEDV N and further investigations are needed to better understand nucleocytoplasmic transport process against PEDV infection.

Proteins belonging to the heat shock protein (HSP) family play a role as chaperones in protein folding. These proteins play similar roles in maintaining proper folding and stabilization of viral proteins [37]. During viral infections, the host cell becomes stressed, and the cellular stress leads to an unfolded protein response (UPR) [38,39]. Our study also shows the enrichment of the spliceosome pathway. Various heterogeneous nuclear ribonucleoproteins, hnRNPA2B1, hnRNPC and hnRNPU, have been confirmed to bind to the PEDV N protein (Figure 5). Spliceosome complex proteins are required to generate stable RNA structures, and ribonucleoproteins play a role in RNA stability [40]. Previous reports showed that hnRNPA2B1 and hnRNPC are associated with influenza A virus (IAV) [41,42], human immunodeficiency virus type 1 (HIV-1) [43], herpes simplex virus 1 (HSV-1) [44,45], hepatitis delta virus (HDV) [46], dengue virus (DENV) [47], and Japanese encephalitis virus (JEV) infection [48], while hnRNPU was a nuclear sensor for viral RNA functions [49]. Herein, we demonstrated that YTHDC1 and NCL relocated from the nucleus/nucleolus to the cytoplasm and inhibited PEDV replication in response to PEDV infection (Figure 6 and Figure 7), inferring that YTHDC1 and NCL had influence on PEDV infection and the precise molecular mechanisms remain unclear and need further study.

In this study, PEDV N–cellular protein interactions in infected Vero cells were identified for the first time. A protein–protein interaction network was constructed, and the potential functions of the characterized cellular proteins were predicted via GO and KEGG enrichment analyses. Eleven selected proteins interacted with the PEDV N protein, as can be seen from the results of the Co-IP assays. The results from the interactions of PEDV N with cellular proteins and the interpretation of the virus–host interaction network may help contribute to a better understanding of the putative mechanisms by which PEDV exerts its pathogenic effects. In addition, the findings from this study also suggest that the replication mechanism and pathogenesis of PEDV are diverse, which means that they need further studies to fully understand them. Increasing our understanding of cellular target proteins and PEDV N protein interference pathways can contribute to a comprehensive understanding of virus–host interactions and provide novel insights for identifying new targets. Ultimately, this information will aid in the development of better therapeutic strategies against PEDV infection.

## Figures and Tables

**Table 1:** List of primers used for cloning in the study.

Gene Product	Sense Primer (5′ to 3′)	Antisense Primer (5′ to 3′)
*PEDV N*	ATGGCTTCTGTCAGTTTTCAGGAT	TTAATTTCCTGTGTCGAAGATCT
*TRIM21*	ATGGCTTCAGCAGCACGCTTGACAA	TCAATAGTCAGTGGATCCTTGTGATC
*DDX24*	ATGAAGTTGAAGGACACAAAATCAAG	TTAATTTGCACTTGTACTTGGCTGTG
*G3BP1*	ATGGTGATGGAGAAGCCTAGTCCCC	TCACTGCCGTGGCGCAAGCC
*HSPA8*	ATGTCCAAGGGACCTGCAGTTGGTAT	TTAATCAACCTCTTCAATAGTGGGCC
*HSP90AB1*	ATGCCTGAGGAAGTGCACCATGGAGA	CTAATCGACTTCTTCCATGCGAGACG
*YTHDC1*	ATGGCGGCCGACAGTCGGGAGGA	TTATCTTCTATATCGACCTCTCTCC
*NCL*	ATGGTGAAGCTCGCGAAGGCAGGTA	CTATTCAAACTTCGTCTTCTTTCCTT
*YBX1*	ATGAGCAGCGAGGCCGAGACCCA	TTACTCAGCCCCGCCCTGCTCAGC
*Vimentin*	ATGACCACCAGGTCCGTGTCCTCGT	TTATTCAAGGTCATCGTGATGCTGAG
*hnRNPA2/B1*	ATGGAGAAAACTTTAGAAACTGTTCC	TCAGTATCGGCTCCTCCCACCATAA
*KPNA1*	ATGACCACCCCAGGAAAAGAGAACTT	TCAAAGCTGGAAACCTTCCATAGGAGC

## Data Availability

All datasets generated for this study are included in the article/Supplementary Material.

## Supplementary Materials

The following supporting information can be downloaded at: https://www.mdpi.com/article/10.3390/v14102269/s1, Table S1: The PEDV nucleocapsid protein-interacting cellular proteins in the PEDV infected (743) and mock infected (500) PK-15 cells, respectively; Table S2: The unique PEDV infected-interacting host proteins (426); Table S3: The GO annotation analyses of PEDV N-interacting host proteins; Table S4: The KEGG enrichment analyses of PEDV N-interacting host proteins; Table S5: The interactions network of the confirmed host protein-PEDV N interaction and the cellular binding partners of PEDV N-interacting host proteins.
